# Comparative Analysis of Volatile Fingerprints and Aroma-Active Compounds in Green and Red Sichuan Pepper Essential Oils Using GC-IMS and GC-O-MS

**DOI:** 10.3390/molecules31152644

**Published:** 2026-07-29

**Authors:** Chu Ding, Xueqian Guo, Yuan Liu, Zhanjiao Wei, Yuhan Zhang, Wei Gao, Changhua Xu, Wenli Wang

**Affiliations:** 1 College of Food Science and Technology, Shanghai Ocean University, Shanghai 201306, China; dingchu18046872894@163.com; 2 Department of Food Science & Technology, School of Agriculture & Biology, Shanghai Jiao Tong University, Shanghai 200240, China; guoxueqian@sjtu.edu.cn (X.G.);; 3 School of Food Science and Engineering, Ningxia University, Yinchuan 750021, China; 4 Chenguang Biotech Group Co., Ltd., Handan 057250, China

**Keywords:** Sichuan pepper essential oil, HS-GC-IMS, GC-O-MS, volatile fingerprint, aroma-active compounds

## Abstract

Green (*Zanthoxylum schinifolium*, ZSEO) and red (*Zanthoxylum bungeanum*, ZBEO) Sichuan pepper essential oils are widely used spices whose characteristic aromas differ markedly, yet a systematic comparison of their volatile fingerprints and aroma-active compounds is still lacking. To address this, the two essential oils were characterized and compared by combining headspace gas chromatography–ion mobility spectrometry (HS-GC-IMS) and gas chromatography–olfactometry–mass spectrometry (GC-O-MS). HS-GC-IMS detected 94 volatile signals; multivariate analysis clearly separated the two oils (R^2^Y = 0.993, Q^2^ = 0.986) and identified 40 differential markers including 37 aroma-active compounds, with ZSEOs enriched in minty and green-note compounds like isomenthone and ZBEOs in woody, ester and acid compounds such as bornyl acetate. GC-O-MS identified 37 aroma-active compounds, of which linalool was the most prominent odorant in both Eos; a total of 27 were shared, while four and six were unique to the green and red EOs, respectively. Cross-referencing the two datasets showed that volatile abundance and odor contribution were not always concordant. The combined approach clarifies the chemical basis of the aroma difference between the two species and offers a transferable strategy for linking volatile fingerprints to perceived aroma in *Zanthoxylum* essential oils.

## 1. Introduction

Sichuan pepper, derived from the pericarps of *Zanthoxylum bungeanum* Maxim. (red Sichuan pepper) and *Zanthoxylum schinifolium* Sieb. et Zucc. (green Sichuan pepper) within the family Rutaceae, is one of the most commercially significant spices in China [1,2]. The characteristic mala (numbing) and aromatic flavors of Sichuan pepper arise from a complex matrix of volatile organic compounds (VOCs) and non-volatile alkylamides, and it is principally the VOC profile that defines the distinctive aroma perceived by consumers [1]. Driven by the rapid expansion of Sichuan-style cuisine globally and the growing demand for natural flavor ingredients in the food industry, Sichuan pepper essential oils (EOs), valued for their concentrated and standardizable aroma, have attracted substantial interest from both academia and the food processing sector [3]. Therefore, a thorough characterization and discrimination of the volatile profiles of the two primary commercial species, *Z. bungeanum* (ZB) and *Z. schinifolium* (ZS), is essential for species quality control, species authentication, and rational flavor design [4].

Previous studies have established that the essential oils of *Z. bungeanum* and *Z. schinifolium* differ substantially in chemical composition. Yang et al. [1] reported that linalyl acetate, linalool, and limonene are the dominant compounds in ZBEO, whereas linalool, limonene, and sabinene predominate in ZSEO. Subsequent investigations using gas chromatography–mass spectrometry (GC-MS) have consistently identified monoterpene hydrocarbons, oxygenated monoterpenes, and sesquiterpenes as the predominant chemical classes in both species, with terpenoids collectively accounting for the majority of the volatile fraction [5,6,7,8,9]. However, conventional GC-MS is limited in that it provides only structural identification and does not convey information about the olfactory relevance of detected compounds [10]. Moreover, in complex essential oil matrices, low-abundance aroma-active compounds with potent odor characteristics may be overlooked [11], compositional data alone provide limited insight into the volatiles responsible for the perceived aromatic distinction between the two species. Gas chromatography–olfactometry–mass spectrometry (GC-O-MS) integrates trained panelists into the analytical workflow, enabling the simultaneous identification and sensory evaluation of eluting compounds [12]. This technique has proven particularly valuable in characterizing aroma-active compounds in Sichuan pepper and related products. Ni et al. [12] employed GC-O with aroma extract dilution analysis (AEDA) and odor activity values (OAVs) to identify key odorants in fried red and green Sichuan pepper oils, establishing that linalool, linalyl acetate, D-limonene, sabinene, and β-myrcene were the primary aroma contributors; Ma et al. [11] applied GC-O-MS in conjunction with GC × GC-TOF-MS to delineate 32 major aroma-active compounds in Sichuan pepper oleoresins from different geographical origins. Wang et al. [13] further characterized the aroma-active compounds in *Z. schinifolium* specifically. Nevertheless, a systematic GC-O-MS comparison of the aroma-active compounds between the EOs of the two major commercial species under equivalent analytical conditions remains limited in the published literature [14,15]. Headspace gas chromatography–ion mobility spectrometry (HS-GC-IMS) has emerged as a powerful complementary technique for volatile profiling. Compared with conventional GC-MS, it operates under ambient conditions with rapid analysis, and high sensitivity for trace-level VOCs [10,16]. It has been increasingly applied to the volatile characterization and authentication of food matrices, including spices [17,18], tea [19,20], fermented beverages [21], and plant-derived oils [22,23]. In Sichuan pepper, Wu et al. [6] applied HS-GC-IMS in combination with HS-SPME-GC-MS to discriminate the volatile profiles of *Z. bungeanum* pericarps from different production regions. Feng et al. [24] further extended this approach to eight geographical indication varieties of Sichuan pepper. Chen et al. [25] employed GC-IMS alongside GC-MS and OAV analysis to investigate the influence of particle size on the volatile profile of Sichuan pepper oil. These studies collectively demonstrate the suitability of GC-IMS for volatile fingerprinting of Sichuan pepper materials; however, its application to the direct comparison of ZBEOs and ZSEOs remains underexplored.

While GC-IMS provides rapid, high-throughput volatile fingerprinting, it is limited by an incomplete spectral library and reduced capability for the structural identification of novel compounds. Conversely, GC-O-MS offers direct sensory relevance but lacks the throughput and two-dimensional resolution of GC-IMS [22,26]. Recent studies have demonstrated that the parallel use of GC-IMS and GC-O/MS constitutes a complementary and mutually validating analytical framework, that has been successfully applied to characterize VOC profiles in pandanus leaves [27], fenugreek tinctures [26] and walnut oil [22], yielding a more complete characterization than either technique alone. However, no study has combined the complementary strengths of HS-GC-IMS and GC-O-MS to systematically characterize and compare the volatile fingerprints and aroma-active compounds of green and red Sichuan pepper essential oils.

To address this gap, the present study aimed to: (1) establish and compare the two-dimensional volatile fingerprints of ZSEOs and ZBEOs by HS-GC-IMS, supported by multivariate statistical analysis including principal component analysis (PCA); (2) identify aroma-active volatile compounds in both essential oils by GC-O-MS and characterize their sensory attributes; and (3) integrate the results from both analytical platforms to provide a comprehensive comparative description of the aroma profiles of green and red Sichuan pepper essential oils. The findings are expected to contribute to the scientific basis for quality differentiation, flavor characterization, and potential industrial applications, like the standardization of commercial EOs, of these two commercially important essential oils.

## 2. Results and Discussion

### 2.1. Volatile Fingerprint Characterization and Multivariate Discrimination of ZSEOs and ZBEOs by HS-GC-IMS

#### 2.1.1. Volatile Composition and Fingerprint Comparison

**Figure 1:**
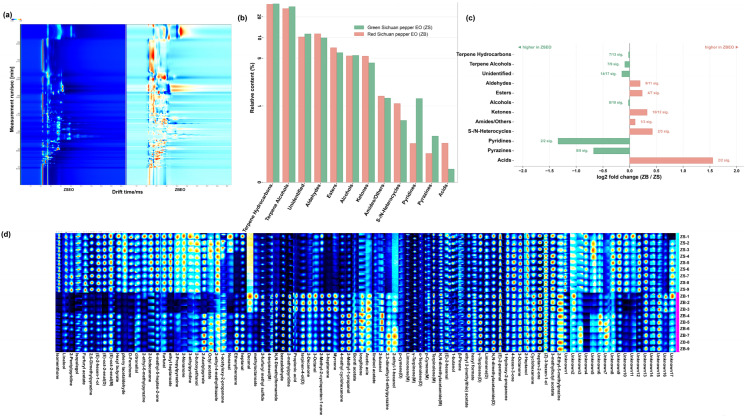
Volatile composition and fingerprints of green (ZSEO, *Zanthoxylum schinifolium*) and red (ZBEO, *Zanthoxylum bungeanum*) Sichuan pepper essential oils analyzed by HS-GC-IMS (*n* = 9 biological replicates per species). (**a**) Difference fingerprints of ZSEO and ZBEO, which red and blue regions denote signals more and less abundant than the reference, respectively, the horizontal axis represents the IMS drift time, and the vertical axis the GC retention time. (**b**) Relative content of each volatile compound class in the two essential oils, expressed as the percentage of total peak volume. (**c**) Between-species difference of each compound class expressed as log_2_ fold change (ZB/ZS), where positive (red) and negative (green) values indicate classes more abundant in ZBEO and ZSEO, respectively. (**d**) Gallery Plot of all characteristic signals, in which each row corresponds to an individual sample (nine replicates per species, ZS-1–9 and ZB-1–9) and each column to a volatile compound, the signal intensity increases from blue to red.

HS-GC-IMS was employed to comprehensively profile the VOCs of ZSEOs and ZBEOs. A total of 94 characteristic signal peaks were detected across both essential oils, of which 77 peaks were identified by matching retention indices and reduced ion mobility values via the G.A.S. IMS library (Table 1) [6,28]. Consistent with the well-established characterization of *Zanthoxylum* volatiles, the identified compounds were dominated by terpene-derived constituents: terpene hydrocarbons (13 compounds) and terpene alcohols and other oxygenated terpenes (9 compounds) together constituted the most abundant chemical classes, accounting for approximately 59% and 57% of the total relative peak volume in ZSEOs and ZBEOs, respectively (Figure 1b) [1,29], followed by aldehydes, esters, alcohols, and ketones. Although the two essential oils shared this broadly similar terpene-dominated framework, clear between-species differences were evident, particularly among the minor compound classes (Figure 1c). The two terpene classes that constitute the bulk of the volatile fraction differed only marginally in relative abundance between the species, whereas several low-abundance classes diverged substantially: acids and aldehydes were proportionally more abundant in ZBEOs, while pyridines and pyrazines were markedly higher in ZSEOs. At the class level, 71 of the 94 peaks were significantly different between the two essential oils after FDR correction (*p* < 0.05), indicating extensive compositional divergence [24].

These differences were directly visualized in the two-dimensional fingerprints. In Figure 1a, numerous signals in the drift-time region were clearly more intense in ZBEO than in ZSEO, confirming that the volatile profiles of ZSEOs and ZBEOs are readily distinguishable. Figure 1d further shows that the nine replicates of each essential oil exhibited highly consistent signal patterns, confirming the reproducibility of the analytical workflow, while the ZS and ZB groups formed two visually distinct blocks. Several compounds, including isomenthone, D-fenchone, isopulegol, 2-ethyl-6-methylpyrazine, and 3-ethylpyridine, which are commonly associated with minty, cooling, herbal, and roasted sensory attributes [30,31], displayed higher signal intensities in ZSEO, whereas bornyl acetate, decanal, longifolene, acetic acid, 2,3-dimethyl-5-ethylpyrazine, and 2-ethyl-1-hexanol, which are connected with woody, floral, fatty, and resinous attributes, were more abundant in ZBEO [32]. These compounds contributed significantly to the discrimination between these two essential oils. In addition, monoterpenes and oxygenated monoterpenes, such as linalool, limonene, β-pinene, γ-terpinene, and terpinen-4-ol, were detected at relatively high levels in both samples, confirming that terpenoids constitute the dominant volatile fraction of Sichuan pepper essential oils.

#### 2.1.2. Multivariate Discrimination and Differential Marker Screening

**Figure 2:**
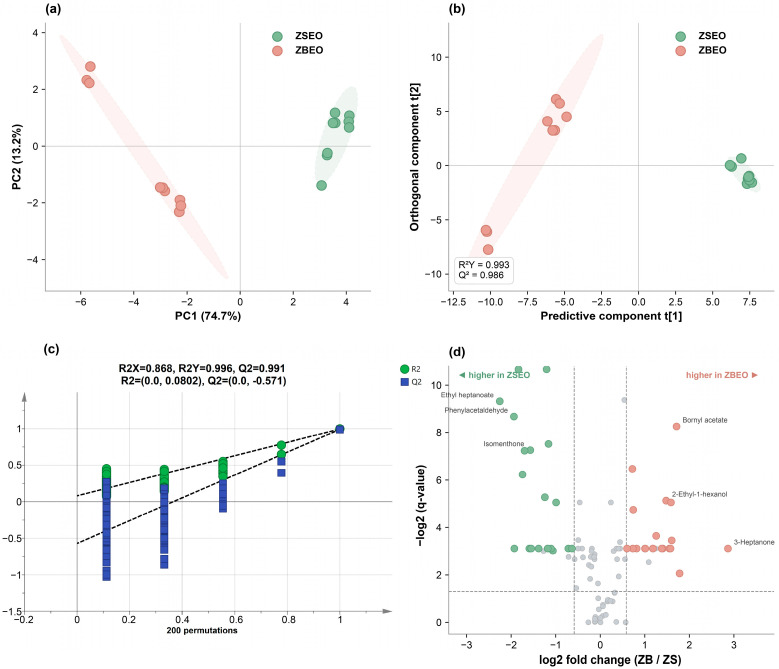
Multivariate discrimination and screening of differential volatile markers between ZSEO and ZBEO based on HS-GC-IMS data (*n* = 9 per group). (**a**) Principal component analysis (PCA) score plot, with the percentage of variance explained given on each axis. (**b**) Orthogonal partial least squares discriminant analysis (OPLS-DA) score plot (R^2^Y = 0.993, Q^2^ = 0.986), where R^2^Y and Q^2^ indicate the goodness of fit and predictive ability of the model, respectively. (**c**) Permutation test (200 iterations) validating the OPLS-DA model, R^2^ < 0.4, Q^2^ < 0.05, confirming the model is not overfitted. (**d**) Volcano plot of all 94 detected peaks; colored points denote the 40 differential markers satisfying VIP > 1, FDR-adjusted *p* < 0.05 and |log_2_(ZB/ZS)| > log_2_1.5, with green and red indicating enrichment in ZSEO and ZBEO, respectively.

To objectively evaluate the separation between the two essential oils, principal component analysis (PCA) was first performed on the GC-IMS peak-volume matrix (Figure 2a). The first two principal components together explained 87.9% of the total variance (PC1 = 74.7%, PC2 = 13.2%), and the two essential oils were clearly separated along PC1. This spontaneous separation, obtained without any prior class information, confirms that species identity is the dominant source of variation in the volatile dataset [18,33]. The supervised OPLS-DA model (Figure 2b) reinforced this separation, resolving the two essential oils completely along the predictive component, the model was statistically reliable and free of overfitting (R^2^Y = 0.993, Q^2^ = 0.986; permutation Q^2^ intercept = −0.50, Figure 2c). The clear, symmetrical displacement of the two groups to opposite sides of the score plot indicates that the two Sichuan pepper essential oils are not merely quantitatively different but systematically different in their volatile composition, such that an unknown sample could in principle be assigned to its species from its HS-GC-IMS fingerprint alone, a discriminating power that has similarly been exploited to distinguish *Zanthoxylum* samples of different varieties and origins [24,29].

The volcano plot was employed to translate this global separation into individual differential markers [34,35]: of the 94 peaks, 40 met the combined screening criteria, these were evenly split between the two oils (Table 1, details in Table A1). The two species were therefore distinguished not by a handful of compounds but by a broad, balanced redistribution of their volatiles. The clearest separation is seen at the extremes of the plot (Figure 2d), where ethyl heptanoate, phenylacetaldehyde, and 2-ethyl-6-methylpyrazine are strongly ZSEO-enriched, while 3-heptanone, bornyl acetate, and propionic acid are most strongly ZBEO-enriched. It is also notable that eight of the markers were unidentified peaks; these peaks (Unknown 1 to Unknown 17) gave reproducible retention time × drift time signals without a match in the G.A.S. IMS library, and were retained as markers based on their statistical values (VIP, *p* and fold change) rather than chemical identity [6,10,18].

Overall, the markers describe two chemically coherent aroma types rather than a random scatter of differences. ZSEO concentrates minty and herbaceous terpenoids (isomenthone, D-fenchone) [36], green-note alcohols ((E)-2-hexen-1-ol) [37], and roasted-smelling pyrazines [38], a combination consistent with its characteristically fresh, clean odor; isomenthone alone accounts for much of the separation, being both the most abundant marker and one of the most divergent (11.37% in ZSEOs versus 3.83% in ZBEOs), which explains its dominant pull on PC1. ZBEO, in contrast, is defined by woody-camphoraceous esters and sesquiterpenes (bornyl acetate, longifolene) [39,40], fatty aldehydes (decanal, benzaldehyde), and short-chain acids (acetic and propionic acid) [6], a profile that matches its heavier, sweeter and slightly sour aroma. The same families of compounds—oxygenated terpenes, esters, aldehydes and acids—have repeatedly been reported as the principal drivers of aroma differences among *Zanthoxylum* species, supporting the reliability of the present markers [1,7,12].

However, the differential markers identified above reflect relative abundance rather than perceived aroma. To establish which of these volatiles are genuinely aroma-active and how they shape the sensory difference between the two essential oils, the same samples were further examined by GC-O-MS [41,42].

### 2.2. Identification and Sensory Characterization of Aroma-Active Compounds in ZSEOs and ZBEOs by GC-O-MS

**Figure 3:**
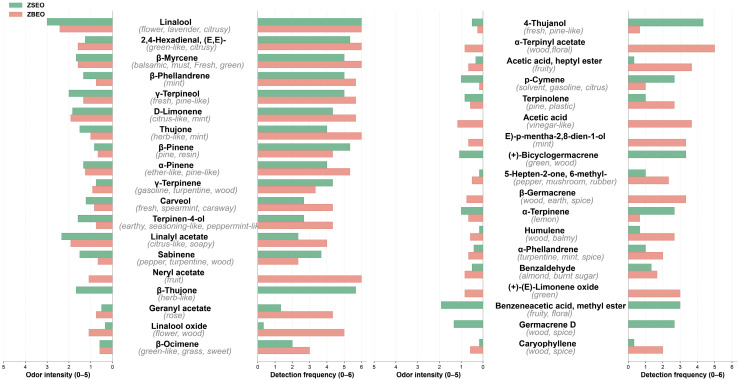
The GC-O-MS results of aroma-active compounds across two species. The left panel presents the odor intensity of the aroma-active compounds in different varieties (0–5, where 0 = not perceived and 5 = extremely strong, averaged over the six assessors), and the right panel shows detection frequency among panelists (6 panelists who perceived each compound). The green and red bars represent ZSEO and ZBEO, respectively, and the compounds are ordered by combined detection frequency. The odor descriptors in parentheses indicate the characteristic sensory notes of each compound.

Where HS-GC-IMS provided a comprehensive overview of the volatile composition, GC-O-MS was employed to identify the volatiles that are directly perceived by the human nose and thus contribute to the characteristic aroma of the two essential oils [41]. Through a synchronous sniffing of the column effluent by six trained assessors, a total of 37 aroma-active compounds were detected across ZSEOs and ZBEOs; the details are shown in Table 2, and the corresponding sensory profiles are visualized in Figure 3 [14,43]. The aroma-active compounds spanned a wide range of chemical classes, with terpene hydrocarbons and oxygenated terpenes again predominating, accompanied by several esters like linalyl acetate, neryl acetate, geranyl acetate, α-terpinyl acetate, a sesquiterpene fraction such as humulene, caryophyllene, germacrene D, and a small number of aldehydes, ketones, and acids. This pattern is consistent with the previous olfactometric studies of Sichuan pepper, in which terpenoids and their oxygenated derivatives were repeatedly identified as the principal odor [11,12,44].

Among all the detected odorants, linalool was the most prominent aroma-active compound in both essential oils, being perceived by all six assessors and exhibiting the highest odor intensity in each sample (3.0 in ZSEOs and 2.4 in ZBEOs), which is in agreement with its consistently reported role as a dominant floral-citrus odorant in *Zanthoxylum* species [12,45,46]. Other compounds shared by both essential oils with high detection frequencies included β-myrcene (balsamic, green), 2,4-hexadienal (green, citrusy), β-phellandrene (mint), D-limonene (citrus, mint), γ-terpineol (fresh, pine-like), and linalyl acetate (citrus, soapy), which together constitute the common aromatic backbone of the two species’ essential oils. In total, 27 of the 37 aroma-active compounds were detected in both essential oils, indicating that the two species share a broadly similar qualitative aroma framework (Figure 3) [14,47].

Despite this shared backbone, marked qualitative and quantitative differences were observed between the two essential oils, providing a sensory-level explanation for their distinct aroma characters (Figure 3). For instance, β-thujone was perceived by almost all assessors in the ZSEOs (frequency 5.7) but by none in the ZBEOs, whereas neryl acetate showed the opposite pattern, reaching the maximum frequency of 6.0 in the ZBEOs while being entirely absent from the ZSEOs. Four aroma-active compounds were detected exclusively in the ZSEOs, which were β-thujone (herb-like), germacrene D (woody, spicy), (+)-bicyclogermacrene (green, woody), and methyl phenylacetate (fruity, floral). Whereas six were unique to the ZBEOs, namely neryl acetate (fruity), α-terpinyl acetate (woody, floral), (E)-p-mentha-2,8-dien-1-ol (mint), (+)-(E)-limonene oxide (green), β-germacrene (woody, earthy), and acetic acid (vinegar-like). The presence of β-thujone together with the higher intensities of mint- and herb-associated odorants in the ZSEOs is consistent with the fresh, green, and minty aroma generally attributed to ZSEOs [1,14]. In contrast, the ZBEOs were characterized by a higher abundance of fruity and floral acetate esters (neryl acetate, α-terpinyl acetate, geranyl acetate, heptyl acetate), an exclusive acetic-acid (sour) note, and a more pronounced woody sesquiterpene contribution (humulene, caryophyllene, β-germacrene), which collectively underlie the heavier, sweeter, and more rounded aroma typical of ZBEOs [12,24,48,49].

Several compounds also displayed clear quantitative shifts between the two species even when present in both. Terpinen-4-ol, sabinene, and methyl phenylacetate showed higher detection frequencies and intensities in ZSEOs, whereas carveol, geranyl acetate, linalool oxide, and terpinolene were more frequently perceived in ZBEOs. These olfactometric trends are in good agreement with the differential markers identified by HS-GC-IMS, where ZSEO was enriched in minty and green-note compounds and ZBEO in woody and ester-type compounds, demonstrating the complementary nature of the two analytical tools [5,11].

While HS-GC-IMS provides information on the relative abundance of volatile compounds, GC-O-MS highlights those that actively contribute to aroma perception. The overlap between the two datasets offers an opportunity to assess whether highly abundant compounds are necessarily aroma-active and to identify volatiles that play important sensory roles despite their relatively low concentrations. Therefore, the common compounds detected by both techniques were further examined.

### 2.3. Integrated Comparison of Aroma Profiles Between ZSEOs and ZBEOs

HS-GC-IMS and GC-O-MS interrogate the volatile fraction from two complementary perspectives: the former provides a comprehensive, instrument-based fingerprint of compound abundance, whereas the latter captures only those volatiles that are actually perceived by the panelists. As the two platforms detect volatiles by different principles, after cross-referencing the two datasets, 13 compounds were detected by both techniques, providing a direct basis for cross-validation (Table 3). For the majority of these shared compounds, the two techniques pointed in a consistent direction. For instance, terpinen-4-ol (earthy, peppermint-like) was substantially more abundant in ZBEOs than in ZSEOs, identified by HS-GC-IMS (4.10% vs. 1.55%), and was correspondingly perceived more frequently at the olfactory port in ZBEOs (detection frequency 4.3 vs. 2.7); benzaldehyde (almond) showed the same concordant pattern (4.35% vs. 2.47% identified by GC-IMS; frequency 1.7 vs. 1.3 identified by GC-O-MS). Such agreement between an instrument-based abundance measure and an independent sensory measure reinforces the reliability of the differential markers identified above [22,27]. More importantly, the joint analysis also revealed cases in which compound abundance and odor contribution were decoupled, underscoring why neither technique alone is sufficient. Linalool is the most striking discovery: although its relative content measured by HS-GC-IMS was only moderate and similar between the two essential oils (1.64% in ZSEOs vs. 1.51% in ZBEOs), it was the single most important odorant in both, being perceived by all six assessors with the highest odor intensity of any compound. Conversely, terpinolene was the most abundant of the shared compounds identified by HS-GC-IMS (8.51% and 7.47%), yet it was only weakly and infrequently perceived (frequency 1.0 and 2.7). This divergence reflects the fact that the sensory impact of a volatile is governed not by its concentration alone. Odor thresholds vary by several orders of magnitude among compounds, such that a constituent of low relative abundance may nonetheless elicit a strong olfactory response when its threshold is correspondingly low [50]. Yu et al. [51] further demonstrated that the addition of terpene alcohols lowered the olfactory threshold of a terpene recombination and exerted a synergistic effect on its herbal and green notes while masking the fruity note, indicating that the terpene matrix itself modulates both the release and the perception of individual odorants. Consistent with this, Grosch [42] reported that odorants with high OAVs are not invariably the principal contributors to an aroma, since certain compounds are suppressed within mixtures whereas others of lower activity values acquire perceptual significance [52]. Irrespective of the underlying mechanism, this decoupling demonstrates the necessity of coupling an abundance-based technique such as GC-IMS with a perception-based technique such as GC-O-MS to obtain a faithful description of an aroma [12,53].

**Figure 4:**
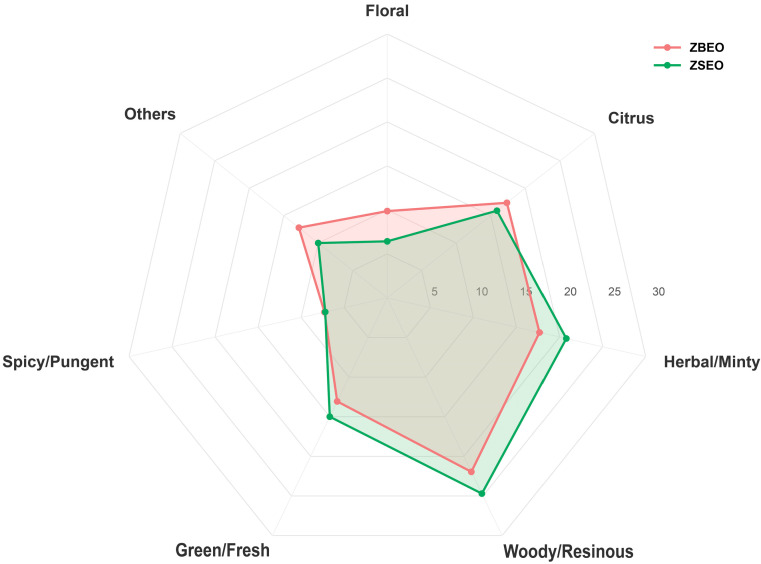
Radar plot comparing the overall aroma profiles of ZSEOs and ZBEOs based on the result of GC-O-MS (*n* = 6 assessors). The 37 aroma-active compounds were grouped into seven aroma dimensions (floral, citrus, herbal/minty, woody/resinous, green/fresh, spicy/pungent, and others) according to their odor descriptors, and the value of each dimension is expressed as its detection frequency normalized to the total detection frequency of the corresponding essential oil (% of total). The green and red areas represent ZSEO and ZBEO, respectively, and a larger area on a given axis indicates a greater relative contribution of that aroma dimension.

To summarize the overall sensory character of the two essential oils, the aroma-active compounds were grouped into seven aroma dimensions according to their descriptors (Figure 4). The two species’ essential oils shared a broadly comparable overall shape, dominated by woody/resinous, herbal/minty, and citrus aromas, which is consistent with the terpene-dominated composition revealed by both techniques. However, clear differences in emphasis were evident. The ZSEOs showed a relatively greater contribution from the herbal/minty and green/fresh dimensions, in line with the exclusive presence of β-thujone and the higher intensities of mint- and green-associated odorants (β-phellandrene, 4-thujanol, sabinene, terpinen-4-ol) observed by GC-O-MS, and with the enrichment of minty markers (isomenthone 11.37% vs. 3.83%, D-fenchone 0.50% vs. 0.17%) detected by HS-GC-IMS [1,14].

In contrast, ZBEOs exhibited a proportionally greater contribution from the floral, citrus, and other dimensions. This pattern is underpinned by the exclusive detection of fruity-floral acetate esters (neryl acetate 1.51% vs. 0.46%, α-terpinyl acetate) and of acetic acid in the ZBEOs, together with the higher abundance of woody-camphoraceous markers (bornyl acetate, longifolene) and fatty aldehydes (decanal, benzaldehyde) identified by HS-GC-IMS. Therefore, the two analytical methods converge on a coherent chemical explanation for the well-recognized sensory distinction between the species: the fresh, green, and minty character of ZSEO arises from its enrichment in mint- and green-note terpenoids, whereas the heavier, sweeter, and more rounded aroma of ZBEO reflects its higher proportion of floral-fruity esters, as well as sour and woody constituents [1,5,12].

Overall, the two techniques converged on a consistent result for the two species. Although green and red Sichuan pepper share a common terpene-dominated framework, green Sichuan pepper (ZS) is distinguished by mint and green note terpenoids that underpin its fresh, herbaceous aroma, whereas red Sichuan pepper (ZB) is characterized by floral-fruity esters, woody sesquiterpenes, and sour acids that give it its heavier, sweeter odor. The agreement between the abundance-based and perception-based results confirms that this chemical contrast is the basis of the well-recognized sensory distinction between the two species [10,22,27].

## 3. Materials and Methods

### 3.1. Samples and Reagents

Green Sichuan pepper essential oil (ZSEO) originating from Jinyang County, Sichuan Province, China, and red Sichuan pepper essential oil (ZBEO) originating from Wudu County, Gansu Province, China, were provided by Chenguang Biotech Group Co., Ltd. (Handan, China). Both EOs were produced by supercritical CO_2_ (SC-CO_2_) extraction at the supplier’s facility; detailed extraction parameters were not disclosed by the supplier and that is a limitation of applicability. Nine independent biological replicates from nine independent production batches were prepared for each species. All samples were stored at −20 °C in the dark prior to analysis.

Additionally, 20 mL headspace vials with 18 mm magnetic PTFE/silicone caps were purchased from ANPEL Laboratory Technologies Inc. (Shanghai, China). Linalool (99.0%), and β-elemene (98%) were purchased from Shanghai Aladdin Biochemical Technology Co., Ltd. (Shanghai, China). γ-terpinene (GC grade), terpinolene (95%), α-terpineol (95%), and γ-terpineol (95%) were purchased from Shanghai Yuanye Bio-Technology Co., Ltd. (Shanghai, China). N-alkane (C7–C40) was purchased from ANPEL Laboratory Technologies Inc. (Shanghai, China). A series of six *n*-methyl ketone reference standards, 2-butanone, 2-pentanone, 2-hexanone, 2-heptanone, 2-octanone, and 2-nonanone (analytically pure), were purchased from Shanghai Aladdin Biochemical Technology Co., Ltd. (Shanghai, China).

### 3.2. GC-IMS Analysis

GC-IMS analyses were performed on a FlavourSpec^®^ instrument (G.A.S. Gesellschaft für Analytische Sensorsysteme mbH, Dortmund, Germany) equipped with a CTC-PAL 3 static headspace autosampler (CTC Analytics AG, Zwingen, Switzerland). Chromatographic separation was performed on an MXT-WAX capillary column (30 m × 0.53 mm × 1.0 μm).

A total of 80 μL of each essential oil sample was placed into a 20 mL headspace vial and incubated at 60 °C for 15 min with 500 rpm. Subsequently, 500 μL of headspace gas was injected into the inlet at 80 °C in splitless mode. High-purity helium (≥99.999%) was used as the carrier gas with the following programmed flow: 2.0 mL/min held for 2 min, linearly increased to 10.0 mL/min over 8 min, then linearly increased to 100.0 mL/min over a further 10 min, and finally held at 100.0 mL/min for 40 min. The column was maintained isothermally at 60 °C throughout the run. High-purity nitrogen (N_2_, purity ≥ 99.999%) served as the IMS drift gas at a flow rate of 75.0 mL/min. The drift tube temperature was maintained at 45 °C, the length of the tube was 53 mm, and the electric field strength was 500 V/cm. Analyte ions were generated by a tritium (^3^H), and all analyses were conducted in positive ion mode.

Raw GC-IMS spectra were processed using VOCal software (version 0.4.03; G.A.S., Dortmund, Germany). The Gallery Plot plugin was applied to extract and align signal peaks across all samples, generating a peak intensity matrix based on the characteristic two-dimensional coordinates of each compound (GC retention time × IMS drift time). Compound identification was achieved by matching retention times and reduced ion mobility values (K_0_) against the G.A.S. IMS Library and NIST spectral database. Retention indices (RIs) were calculated from the retention times of a series of six *n*-methyl ketone reference standards (C4–C9). The value of a peaks’ relative abundance was calculated using following formula [54]:

$$Relative\;Abundance(\%)=\frac{v_{i}}{\sum_{i=1}^{n}v_{i}}\times 100\;$$

where $V_{i}$ is the peak volume of the individual compound and $\sum V_{i}$ is the sum of the peak volumes of all identified compounds.

### 3.3. HS-SPME-GC-O-MS Analysis

Aroma-active compounds were analyzed using an Agilent 7890A gas chromatograph (Agilent Technologies, Santa Clara, CA, USA) coupled to an Agilent 7000E mass spectrometer and an ODP4 olfactory detection port (Gerstel GmbH & Co. KG, Mülheim an der Ruhr, Germany). Chromatographic separation was achieved on a DB-WAX capillary column (30 m × 250 μm × 0.25 μm; Agilent J&W, Santa Clara, CA, USA).

A total of 0.170 g of each essential oil sample was accurately weighed into a 20 mL headspace vial, which was immediately sealed. After equilibration at 60 °C for 30 min, a DVB/CAR/PDMS SPME fiber (50/30 μm; Supelco, Bellefonte, PA, USA) was inserted into the headspace of the vial and exposed for 30 min to adsorb volatile compounds. The fiber was subsequently transferred to the GC injector port and thermally desorbed at 250 °C for 5 min. Helium (purity > 99.999%) was used as the carrier gas at a flow rate of 1 mL/min; the injection mode split ratio was 10:1. The temperature program referred to [55] with slight modifications, as follows: 50 °C held for 5 min, followed by an increase to 86 °C at 1.5 °C/min held for 1 min, a further rise to 115 °C at 3.0 °C/min, then an increase to 124 °C at 1.0 °C/min held for 4 min, and finally a ramp up to 240 °C at 15.0 °C/min. The mass spectrometer was operated in electron ionization (EI) mode at 70 eV with a mass scanning range of 30–450 *m*/*z*. The temperatures for the ionization source and transmission line were defined at 230 °C and 250 °C, respectively. The ODP4, with its transfer line maintained at 250 °C, was connected to the GC-MS instrument to detect the sensory attributes of volatile aroma compounds via trained sensory evaluators (three males and three females, 22–30 years old), who were from Shanghai Jiao Tong University (Shanghai, China) and screened and trained in accordance with ISO 13301:2018 [56] and ISO 8586:2012 [57]. Prior to formal analysis, the assessors were trained with a set of reference odorants to familiarize themselves with the olfactory detection port and to establish a common understanding of the aroma descriptors and the intensity scale. During each run, the column effluent was split 1:1 between the mass spectrometer and the ODP4, and each assessor sniffed the entire chromatographic run of both essential oils, recording the retention time, aroma descriptor, and perceived intensity of every detected odor on a six-point scale (0 = not perceived, 1 = very weak, 3 = moderate, 5 = extremely strong). The sample elution order was randomized across assessors to minimize systematic bias. Each oil was evaluated once by each of the six assessors, and a compound was considered aroma-active only when detected by at least two of them. Detection frequency (0–6) was defined as the number of assessors perceiving a given odor, and mean intensity as the corresponding average across assessors, with an agreement between these two independent measures serving as an indicator of panel consistency.

The volatile compounds were qualified by MS characterization, retention index (RI) comparison, and comparing with established standards [43]. The MS results were searched using Agilent MassHunter Qualitative Analysis B.07.00 (Agilent Technologies Inc., Santa Clara, CA, USA) and the NIST20L, the mass spectrum database (National Institute of Standards and Technology, Gaithersburg, MD, USA). The RI value of a target compound was calculated by matching the peak time of it with the peak time of a series of *n*-alkanes (C_7_–C_40_) using the following formula [58]:

$$RI=100\times \left[\;n+\frac{\log_{t_{R}}\left(x\right)-\log_{t_{R}}\left(n\right)}{\log_{t_{R}}\left(n+1\right)-\log_{t_{R}}\left(n\right)}\right]$$

where $t_{R}(x)$ is the retention time of the volatile compound; $t_{R}(n)$ and $t_{R}(n+1)$ are the retention times of the *n*-alkanes eluting immediately before and after the compound, respectively; and n is the carbon number of the *n*-alkane eluting immediately before the compound.

### 3.4. Statistical Analysis

All data are expressed as mean ± standard deviation (SD) of nine replicates (*n* = 9). For each volatile peak, the relative content was compared between the two essential oil types using IBM SPSS Statistics 25.0 (IBM Corp., Armonk, NY, USA). Prior to hypothesis testing, the normality and homogeneity of variance of each peak were assessed by the Shapiro–Wilk test and Levene’s test, respectively; an independent-samples Student’s *t*-test, Welch’s *t*-test, or the non-parametric Mann–Whitney *U* test was then applied accordingly. To account for multiple comparisons across all peaks, the resulting *p*-values were adjusted by the Benjamini–Hochberg false discovery rate (FDR) procedure, and differences with an adjusted *p* < 0.05 were considered statistically significant.

Multivariate statistical analyses, including principal component analysis (PCA) and orthogonal partial least squares discriminant analysis (OPLS-DA), were performed using SIMCA 14.1 (Sartorius Stedim Data Analytics AB, Umeå, Sweden); model quality was assessed by R^2^Y and Q^2^, and validated by a 200-permutation test to exclude overfitting. Volatile compounds simultaneously satisfying a variable importance in projection (VIP) score > 1, an FDR-adjusted *p* < 0.05, and an absolute fold change |log_2_(ZB/ZS)| > log_2_1.5 were defined as robust differential markers between the two essential oil types. Other data visualization was performed using Python (version 3.13; Python Software Foundation, Wilmington, DE, USA) with the pandas (3.0.5), scikit-learn (1.9.0), SciPy (1.18.0), Matplotlib (3.11.1), and Seaborn packages (0.13.2).

## 4. Conclusions

In this study, the volatile fingerprints and aroma-active compounds of green and red Sichuan pepper essential oils were systematically characterized and compared by combining HS-GC-IMS and GC-O-MS. HS-GC-IMS established distinct volatile fingerprints for the two essential oils and identified 40 robust differential markers, demonstrating that the two species differ markedly despite sharing a common terpene-dominated framework. GC-O-MS complemented these instrumental data by linking the chemical differences to perceptible sensory attributes, revealing that the fresh, green, and minty character of ZSEOs and the heavier, woody, and sweeter character of ZBEOs arise from their contrasting profiles of mint/green-note versus woody/ester/acid compounds.

Importantly, the joint analysis showed that volatile abundance does not necessarily reflect odor contribution, underscoring the value of integrating an abundance-based technique with a perception-based one. The combined HS-GC-IMS and GC-O-MS approach thus offers a comprehensive and complementary strategy for elucidating the aroma differences between *Zanthoxylum* species and may provide a useful reference for future quality evaluation and authentication studies. Nevertheless, each species was represented by a single production source. HS-GC-IMS provides relative rather than absolute quantification, and the sensory data were obtained from a small, trained panel rather than by consumer evaluation. Future work incorporating multiple geographical origins, quantitative odor-activity measurements (e.g., AEDA or OAV), and applications to adulterated or differently processed samples would further strengthen the characterization of their aroma profiles.

## Figures and Tables

**Table 1:** Differential volatile markers between green (ZS) and red (ZB) Sichuan pepper essential oils screened by HS-GC-IMS.

Class	Compound	RI	CAS	Abundance (%)		*p*-Value (FDR)	log_2_FC (ZB/ZS)	VIP
				ZS	ZB			
Terpene Hydrocarbon	Longifolene ***	1587.6	475-20-7	0.479 ± 0.010	0.800 ± 0.087	1.81 ×10^−5^	0.741	1.244
	Ethenylbenzene **	1252.2	100-42-5	0.019 ± 0.015	0.064 ± 0.027	8.69 × 10^−3^	1.781	1.007
Terpene Alcohols	Terpinen-4-ol ***	1582.4	562-74-3	1.550 ± 0.213	4.096 ± 0.783	7.75 × 10^−4^	1.402	1.195
	Isopulegol ***	1562.3	89-79-2	1.384 ± 0.056	0.842 ± 0.332	7.75 × 10^−4^	−0.716	1.024
	Fenchone ***	1405.5	4695-62-9	0.497 ± 0.054	0.165 ± 0.041	7.75 × 10^−4^	−1.591	1.252
	Isomenthone ***	1487.2	1196-31-2	11.374 ± 0.222	3.831 ± 0.990	5.56 × 10^−8^	−1.57	1.276
	Bornyl acetate ***	1591.5	76-49-3	0.459 ± 0.043	1.506 ± 0.123	5.54 × 10^−9^	1.713	1.282
Alcohols	2-Butanol ***	1020.3	78-92-2	0.137 ± 0.035	0.275 ± 0.047	7.75 × 10^−4^	1.005	1.154
	2-Methyl-1-propanol ***	1102.7	78-83-1	0.281 ± 0.016	0.825 ± 0.193	7.75 × 10^−4^	1.554	1.215
	2-Ethyl-1-hexanol ***	1482.8	104-76-7	0.010 ± 0.001	0.028 ± 0.005	7.43 × 10^−6^	1.479	1.232
	(E)-2-Hexen-1-ol ***	1410.3	928-95-0	0.439 ± 0.026	0.196 ± 0.055	3.03 × 10^−8^	−1.161	1.229
Aldehydes	Benzaldehyde ***	1520.5	100-52-7	2.467 ± 0.093	4.345 ± 0.212	7.75 × 10^−4^	0.817	1.293
	Phenylacetaldehyde ***	1622.7	122-78-1	0.211 ± 0.016	0.055 ± 0.006	2.14 × 10^−9^	−1.938	1.289
	Decanal ***	1484.1	112-31-2	0.009 ± 0.001	0.019 ± 0.006	7.75 × 10^−4^	1.181	1.074
	(E)-Oct-2-enal ***	1430.8	2548-87-0	0.826 ± 0.068	0.395 ± 0.240	9.67 × 10^−4^	−1.063	1.039
Ketones	4-Methylcyclohexanone ***	1348.8	589-92-4	0.169 ± 0.031	0.383 ± 0.039	7.75 × 10^−4^	1.184	1.255
	2-Nonanone ***	1370.4	821-55-6	0.031 ± 0.003	0.011 ± 0.001	7.75 × 10^−4^	−1.55	1.274
	2-Decanone ***	1480.4	693-54-9	0.086 ± 0.010	0.204 ± 0.047	2.27 × 10^−4^	1.256	1.147
	2-Undecanone ***	1594.6	112-12-3	0.244 ± 0.026	0.113 ± 0.027	7.75 × 10^−4^	−1.106	1.238
	3-Heptanone ***	1148.8	106-35-4	0.054 ± 0.012	0.390 ± 0.091	7.75 × 10^−4^	2.867	1.220
	6-Methyl-5-hepten-2-one ***	1342.9	110-93-0	0.156 ± 0.007	0.100 ± 0.019	7.75 × 10^−4^	−0.635	1.175
Esters	Hexyl butyrate ***	1415.6	2639-63-6	0.163 ± 0.018	0.069 ± 0.031	5.34 × 10^−6^	−1.243	1.157
	Ethyl heptanoate ***	1369.6	106-30-9	0.155 ± 0.019	0.033 ± 0.012	4.86 × 10^−10^	−2.255	1.259
	Ethyl 2-(methylthio)acetate ***	1456.3	4455-13-4	0.381 ± 0.021	0.632 ± 0.067	7.75 × 10^−4^	0.731	1.236
Acids	Acetic acid ***	1466.8	64-19-7	0.024 ± 0.008	0.062 ± 0.009	7.75 × 10^−4^	1.38	1.193
	Propionic acid ***	1508.9	79-09-4.	0.151 ± 0.023	0.451 ± 0.165	7.75 × 10^−4^	1.577	1.061
Pyrazines	2-Propylpyrazine ***	1428.1	18,138-03-9	0.042 ± 0.008	0.018 ± 0.006	7.75 × 10^−4^	−1.18	1.136
	2,6-Dimethylpyrazine ***	1327.7	108-50-9	0.315 ± 0.031	0.139 ± 0.037	7.75 × 10^−4^	−1.178	1.218
	2-Ethyl-6-methylpyrazine ***	1363.1	13,925-03-6	0.116 ± 0.016	0.036 ± 0.014	5.90 × 10^−8^	−1.699	1.229
	2,3-Dimethyl-5-ethylpyrazine ***	1447.5	15,707-34-3	0.008 ± 0.001	0.024 ± 0.004	8.83 × 10^−6^	1.587	1.228
Pyridines	2-Pentylpyridine ***	1572.4	2294-76-0	1.220 ± 0.270	0.468 ± 0.227	7.75 × 10^−4^	−1.383	1.101
Amides/other	N,N-Dimethylformamide ***	1325.7	68-12-2.	0.023 ± 0.003	0.047 ± 0.006	7.75 × 10^−4^	1.011	1.226
Unidentified	Unknown3 ***		-	0.032 ± 0.005	0.053 ± 0.004	3.41 × 10^−7^	0.721	1.217
	Unknown4 ***		-	0.359 ± 0.044	0.107 ± 0.009	5.93 × 10^−7^	−1.745	1.270
	Unknown5 ***		-	0.078 ± 0.014	0.039 ± 0.007	8.83 × 10^−6^	−0.991	1.139
	Unknown7 ***		-	0.014 ± 0.002	0.044 ± 0.012	3.52 × 10^−4^	1.604	1.167
	Unknown9 ***		-	0.283 ± 0.013	0.123 ± 0.019	2.17 × 10^−11^	−1.204	1.275
	Unknown10 ***		-	0.185 ± 0.012	0.052 ± 0.015	2.17 × 10^−11^	−1.835	1.276
	Unknown12 ***		-	0.174 ± 0.031	0.046 ± 0.007	7.75 × 10^−4^	−1.928	1.232
	Unknown15 ***		-	0.483 ± 0.067	0.733 ± 0.021	7.75 × 10^−4^	0.601	1.215

ZS, green (*Z. schinifolium*) and ZB, red (*Z. bungeanum*) Sichuan pepper essential oil. Data are mean ± SD (*n* = 9). *p*, FDR-adjusted *p*-value (*** *p* < 0.001, ** *p* < 0.01). log_2_FC (ZB/ZS): positive, higher in ZB; negative, higher in ZS. VIP, variable importance in projection. All of the robust markers are accorded with (VIP > 1, *p* < 0.05, |log_2_FC| > log_2_1.5).

**Table 2:** Aroma-active compounds identified in ZSEOs and ZBEOs by GC-O-MS.

Compound	Aroma Descriptor	RI	CAS	Frequency		Intensity	
				ZS	ZB	ZS	ZB
**Linalool**	flower, lavender, citrusy	1561.3	78-70-6	6.00	6.00	3.00	2.42
**2,4-Hexadienal, (E,E)-**	green-like, citrusy	1403.8	142-83-6	5.33	6.00	1.25	1.58
**β-Myrcene**	balsamic, must, fresh, green	1170.7	123-35-3	5.00	6.00	1.67	1.58
**β-Phellandrene**	mint	1128.2	555-10-2	5.00	5.67	1.33	0.75
**γ-Terpineol**	fresh, pine-like	1534.7	586-81-2	5.00	5.67	2.00	1.33
**Limonene**	citrus-like, mint	1214.3	5989-27-5	4.33	5.67	1.83	1.92
Thujone	herb-like, mint	1429.9	546-80-5	4.00	6.00	1.50	1.00
β-Pinene	pine, resin	1115.6	127-91-3	5.33	4.33	0.83	0.67
α-Pinene	ether-like, pine-like	1025.1	80-56-8	4.00	5.33	1.33	1.25
γ-Terpinene	gasoline, turpentine, wood	1254.2	99-85-4	4.33	3.33	0.75	0.92
**Carveol**	fresh, spearmint, caraway	1883.2	99-48-9	2.67	4.33	1.22	0.83
**Terpinen-4-ol**	earthy, peppermint-like	1608.1	562-74-3	2.67	4.33	1.58	0.75
**Linalyl acetate**	citrus-like, soapy	1572.1	115-95-7	2.33	4.00	2.33	1.9
**Sabinene**	pepper, turpentine, wood	1224.3	3387-41-5	3.67	2.33	1.50	0.67
**Neryl acetate**	fruity	1734.7	141-12-8	0.00	6.00	0.00	1.08
**β-Thujone**	herb-like	1436.9	471-15-8	5.67	0.00	1.67	0.00
**Geranyl acetate**	rose	1774.8	105-87-3	1.33	4.33	0.50	0.75
**Linalool oxide**	flower, wood	1456.3	60,047-17-8	0.33	5.00	0.33	1.08
β-Ocimene	green-like, grass, sweet	1258.7	13,877-91-3	2.00	3.00	0.58	0.58
**4-Thujanol**	fresh, pine-like	1467.2	546-79-2	4.33	0.67	0.50	0.25
**α-Terpinyl acetate**	wood, floral	1694.7	80-26-2	0.00	5.00	0.00	0.83
Acetic acid, heptyl ester	fruity	1388.3	112-06-1	0.33	3.67	0.33	0.67
*p*-Cymene	solvent, gasoline, citrus	1279.6	99-87-6	2.67	1.00	1.00	0.17
**Terpinolene**	pine, plastic	1283.8	586-62-9	1.00	2.67	0.83	0.58
**Acetic acid**	vinegar-like	1472.2	64-19-7	0.00	3.67	0.00	1.17
**(E)-p-mentha-2,8-dien-1-ol**	mint	1558.3	3886-78-0	0.00	3.33	0.00	0.67
**Bicyclogermacrene**	green, wood	1725.5	24,703-35-3	3.33	0.00	1.08	0.00
5-Hepten-2-one, 6-methyl-	pepper, mushroom, rubber	1391.4	110-93-0	1.00	2.33	0.17	0.50
**β-Germacrene**	wood, earth, spice	1839.5	15,423-57-1	0.00	3.33	0.00	0.75
α-Terpinene	lemon	1168.5	99-86-5	2.67	0.67	1.00	0.67
Humulene	wood, balmy	1678.5	6753-98-6	0.67	2.67	0.17	0.58
α-Phellandrene	turpentine, mint, spice	1031.9	99-83-2	1.00	2.00	0.42	0.67
**Benzaldehyde**	almond, burnt sugar	1519.9	100-52-7	1.33	1.67	0.50	0.83
**(E)-Limonene oxide**	green	1466.6	6909-30-4	0.00	3.00	0.00	0.83
**Benzeneacetic acid, methyl ester**	fruity, floral	1764.3	101-41-7	3.00	0.00	1.92	0.00
**Germacrene D**	wood, spice	1690.4	37,839-63-7	2.67	0.00	1.33	0.00
Caryophyllene	wood, spice	1587.0	87-44-5	0.33	2.00	0.17	0.58

ZS, green and ZB, red Sichuan pepper essential oil. RI, retention index (DB-WAX, C7–C40 *n*-alkanes). Detection frequency (0–6) and odor intensity (0–5) are means of the six assessors (*n* = 6); 0 indicates not perceived.

**Table 3:** Compounds detected by both HS-GC-IMS and GC-O-MS in the two essential oils.

Compound	Relative Abundance		Frequency	
	ZS	ZB	ZS	ZB
Terpinolene	8.51	7.47	1	2.7
Limonene	5.2	5.1	4.3	5.7
β-Pinene	3.78	3.17	5.3	4.3
Benzaldehyde	2.47	4.35	1.3	1.7
Terpinen-4-ol	1.55	4.1	2.7	4.3
*p*-Cymene	2.61	2.53	2.7	1
α-Phellandrene	2.17	2.01	1	2
Linalool	1.64	1.51	6	6
Linalyl acetate	0.9	1.23	2.3	4
γ-Terpinene	0.64	0.85	4.3	3.3
5-Hepten-2-one, 6-methyl-	0.16	0.1	1	2.3
β-Myrcene	0.05	0.12	5	6
Acetic acid	0.02	0.06	0	3.7

Compounds co-detected by HS-GC-IMS and GC-O-MS. GC-IMS relative content, mean % of total peak volume (*n* = 9); GC-O detection frequency (0–6), mean of the six assessors (*n* = 6).

## Data Availability

The original contributions presented in this study are included in the article. Further inquiries can be directed to the corresponding author.

## Abbreviations

The following abbreviations are used in this manuscript:

ZS	Green Sichuan pepper (*Zanthoxylum schinifolium*)
ZB	Red Sichuan pepper (*Zanthoxylum bungeanum*)
EO	Essential oil
ZSEOs	*Zanthoxylum schinifolium* Essential oils
ZBEOs	*Zanthoxylum bungeanum* Essential oils

## Appendix A

**Table A1:** Summary of VOCs detected by GC-IMS.

Class	Compound	RI	CAS	Abundance(%)		q-Value (FDR)	log_2_FC (ZB/ZS)	VIP
				ZS	ZB			
Terpene Hydrocarbon	Myrcene ***	1168.7	123-35-3	0.050 ± 0.006	0.118 ± 0.063	7.75 × 10^−4^	1.245	0.877
	α-Phellandrene	1030.1	99-83-2	2.167 ± 0.216	2.009 ± 0.329	5.93 × 10^−1^	−0.110	0.559
	β-Pinene **	1112.3	127-91-3	3.783 ± 0.263	3.171 ± 0.311	3.88 × 10^−3^	−0.255	1.015
	Limonene(1) ***	1210.7	138-86-3	2.340 ± 0.269	2.881 ± 0.066	9.99 × 10^−4^	0.300	1.071
	Limonene(2)	1210.7	138-86-3	5.198 ± 0.264	5.101 ± 0.245	8.45 × 10^−1^	−0.027	0.389
	p-Cymene(1)	1277.4	99-87-6	0.182 ± 0.009	0.228 ± 0.057	5.79 × 10^−2^	0.322	0.736
	p-Cymene(2)	1277.4	99-87-6	2.609 ± 0.105	2.529 ± 0.053	9.83 × 10^−2^	−0.045	0.588
	γ-Terpinene(1)	1250.8	99-85-4	3.160 ± 0.304	3.285 ± 0.224	5.37 × 10^−1^	0.056	0.346
	γ-Terpinene(2) *	1250.8	99-85-4	0.638 ± 0.195	0.851 ± 0.084	1.17 × 10^−2^	0.415	0.790
	Terpinolene(1) ***	1237.1	586-62-9	1.717 ± 0.064	1.924 ± 0.274	1.33 × 10^−4^	0.164	0.720
	Terpinolene(2) ***	1237.1	586-62-9	8.508 ± 0.100	7.474 ± 0.677	7.75 × 10^−4^	−0.187	0.998
	Longifolene ***	1587.6	475-20-7	0.479 ± 0.010	0.800 ± 0.087	1.81 × 10^−5^	0.741	1.244
	Ethenylbenzene **	1252.2	100-42-5	0.019 ± 0.015	0.064 ± 0.027	8.69 × 10^−3^	1.781	1.007
Terpene Alcohols	Linalool	1560.4	78-70-6	1.645 ± 0.039	1.514 ± 0.330	9.60 × 10^−1^	−0.120	0.568
	Terpinen-4-ol(1) ***	1582.4	562-74-3	10.203 ± 0.369	13.144 ± 1.146	7.75 × 10^−4^	0.365	1.143
	Terpinen-4-ol(2) ***	1582.4	562-74-3	1.550 ± 0.213	4.096 ± 0.783	7.75 × 10^−4^	1.402	1.195
	Isopulegol ***	1562.3	89-79-2	1.384 ± 0.056	0.842 ± 0.332	7.75 × 10^−4^	−0.716	1.024
	Fenchone ***	1405.5	4695-62-9	0.497 ± 0.054	0.165 ± 0.041	7.75 × 10^−4^	−1.591	1.252
	Isomenthone ***	1487.2	1196-31-2	11.374 ± 0.222	3.831 ± 0.990	5.56 × 10^−8^	−1.57	1.276
	Linalyl acetate ***	1570.1	115-95-7	0.902 ± 0.054	1.229 ± 0.100	7.75 × 10^−4^	0.446	1.176
	Bornyl acetate ***	1591.5	76-49-3	0.459 ± 0.043	1.506 ± 0.123	5.54 × 10^−9^	1.713	1.282
	Octyl acetate	1478.3	112-14-1	0.081 ± 0.015	0.074 ± 0.031	9.08 × 10^−1^	−0.12	0.415
Alcohols	1-Butanol ***	1150.8	71-36-3	1.195 ± 0.159	0.902 ± 0.114	7.75 × 10^−4^	−0.407	0.972
	2-Butanol ***	1020.3	78-92-2	0.137 ± 0.035	0.275 ± 0.047	7.75 × 10^−4^	1.005	1.154
	2-Methyl-1-propanol ***	1102.7	78-83-1	0.281 ± 0.016	0.825 ± 0.193	7.75 × 10^−4^	1.554	1.215
	2-Heptanol **	1293.6	543-49-7	1.808 ± 0.116	1.657 ± 0.059	1.35 × 10^−3^	−0.126	0.856
	3-Octanol	1356.8	589-98-0	0.093 ± 0.003	0.113 ± 0.033	1.33 × 10^−1^	0.277	0.648
	2-Ethyl-1-hexanol ***	1482.8	104-76-7	0.010 ± 0.001	0.028 ± 0.005	7.43 × 10^−6^	1.479	1.232
	2-Butoxyethanol **	1416.5	111-76-2	0.093 ± 0.012	0.062 ± 0.014	2.21 × 10^−3^	−0.576	1.007
	(Z)-3-Hexen-1-ol	1385.6	928-96-1	0.602 ± 0.029	0.589 ± 0.027	2.88 × 10^−1^	−0.033	0.348
	(E)-2-Hexen-1-ol ***	1410.3	928-95-0	0.439 ± 0.026	0.196 ± 0.055	3.03 × 10^−8^	−1.161	1.229
	1-Hydroxy-2-propanone **	1301.6	116-09-6	0.810 ± 0.044	0.741 ± 0.032	2.21 × 10^−3^	−0.129	0.923
Aldehydes	Benzaldehyde ***	1520.5	100-52-7	2.467 ± 0.093	4.345 ± 0.212	7.75 × 10^−4^	0.817	1.293
	Phenylacetaldehyde ***	1622.7	122-78-1	0.211 ± 0.016	0.055 ± 0.006	2.14 × 10^−9^	−1.938	1.289
	Decanal ***	1484.1	112-31-2	0.009 ± 0.001	0.019 ± 0.006	7.75 × 10^−4^	1.181	1.074
	Nonanal ***	1394.8	124-19-6	0.013 ± 0.006	0.005 ± 0.002	9.99 × 10^−4^	−1.273	0.841
	Heptanal	1211.6	111-71-7	0.166 ± 0.076	0.208 ± 0.048	5.52 × 10^−2^	0.329	0.488
	(E)-Oct-2-enal(1) ***	1430.8	2548-87-0	0.777 ± 0.032	0.551 ± 0.146	7.75 × 10^−4^	−0.496	1.000
	(E)-Oct-2-enal(2) ***	1430.8	2548-87-0	0.826 ± 0.068	0.395 ± 0.240	9.67 × 10^−4^	−1.063	1.039
	(E)-2-Hexenal **	1222.4	6728-26-3	0.622 ± 0.062	0.542 ± 0.030	4.53 × 10^−3^	−0.199	0.85
	(E)-2-Pentenal	1128.9	1576-87-0	4.184 ± 0.692	4.742 ± 0.306	1.16 × 10^−1^	0.181	0.652
	Furfural ***	1440.3	98-01-1.	0.194 ± 0.014	0.138 ± 0.029	3.40 × 10^−4^	−0.494	1.049
	Citronellal ***	1491.8	106-23-0	0.414 ± 0.023	0.301 ± 0.040	8.83 × 10^−6^	−0.461	1.145
Ketones	Cyclohexanone ***	1305.8	108-94-1	0.081 ± 0.004	0.095 ± 0.005	8.83 × 10^−6^	0.239	1.143
	4-Methylcyclohexanone ***	1348.8	589-92-4	0.169 ± 0.031	0.383 ± 0.039	7.75 × 10^−4^	1.184	1.255
	2-Decanone ***	1480.4	693-54-9	0.086 ± 0.010	0.204 ± 0.047	2.27 × 10^−4^	1.256	1.147
	2-Undecanone ***	1594.6	112-12-3	0.244 ± 0.026	0.113 ± 0.027	7.75 × 10^−4^	−1.106	1.238
	3-Octanone	1271.4	106-68-3	0.547 ± 0.029	0.570 ± 0.011	1.85 × 10^−1^	0.061	0.648
	2-Heptanone ***	1203.5	110-43-0	1.760 ± 0.080	2.338 ± 0.220	7.75 × 10^−4^	0.410	1.148
	3-Heptanone ***	1148.8	106-35-4	0.054 ± 0.012	0.390 ± 0.091	7.75 × 10^−4^	2.867	1.220
	4-Hexen-3-one ***	1198.4	2497-21-4	0.715 ± 0.021	0.625 ± 0.039	9.99 × 10^−4^	−0.194	1.134
	2-Nonanone ***	1370.4	821-55-6	0.031 ± 0.003	0.011 ± 0.001	7.75 × 10^−4^	−1.55	1.274
	6-Methyl-5-hepten-2-one ***	1342.9	110-93-0	0.156 ± 0.007	0.100 ± 0.019	7.75 × 10^−4^	−0.635	1.175
	2-Methyl-2-cyclopenten-1-one **	1360.4	1120-73-6	0.051 ± 0.029	0.108 ± 0.022	2.93 × 10^−3^	1.088	0.996
	Acetol acetate	1477.6	592-20-1	0.368 ± 0.178	0.402 ± 0.072	4.83 × 10^−1^	0.126	0.183
Esters	Furfuryl acetate	1517.9	623-17-8	2.263 ± 0.393	2.081 ± 0.636	7.12 × 10^−1^	−0.121	0.45
	Hexyl butyrate ***	1415.6	2639-63-6	0.163 ± 0.018	0.069 ± 0.031	5.34 × 10^−6^	−1.243	1.157
	Hexyl formate ***	1368.5	112-33-4	2.623 ± 0.145	3.815 ± 0.156	4.29 × 10^−10^	0.541	1.261
	Methyl octanoate	1388.2	111-11-5	0.051 ± 0.005	0.066 ± 0.043	9.60 × 10^−1^	0.360	0.502
	Ethyl heptanoate ***	1349.6	106-30-9	0.155 ± 0.019	0.033 ± 0.012	4.86 × 10^−10^	−2.255	1.259
	3-Methylbutyl acetate	1097.2	123-92-2	0.370 ± 0.044	0.372 ± 0.051	1.00 × 10^0^	0.006	0.298
	Ethyl 2-(methylthio)acetate ***	1456.3	4455-13-4	0.381 ± 0.021	0.632 ± 0.067	7.75 × 10^−4^	0.731	1.236
Acids	Acetic acid ***	1466.8	64-19-7	0.024 ± 0.008	0.062 ± 0.009	7.75 × 10^−4^	1.38	1.193
	Propionic acid ***	1508.9	79-09-4	0.151 ± 0.023	0.451 ± 0.165	7.75 × 10^−4^	1.577	1.061
Pyrazines	2-Propylpyrazine ***	1428.1	18138-03-9	0.042 ± 0.008	0.018 ± 0.006	7.75 × 10^−4^	−1.18	1.136
	2,6-Dimethylpyrazine ***	1327.7	108-50-9	0.315 ± 0.031	0.139 ± 0.037	7.75 × 10^−4^	−1.178	1.218
	2-Ethyl-5-methylpyrazine **	1384.5	13360-65-1	0.124 ± 0.008	0.162 ± 0.025	2.21 × 10^−3^	0.378	0.949
	2-Ethyl-6-methylpyrazine ***	1363.1	13925-03-6	0.116 ± 0.016	0.036 ± 0.014	5.90 × 10^−8^	−1.699	1.229
	2,3-Dimethyl-5-ethylpyrazine ***	1447.5	15707-34-3	0.008 ± 0.001	0.024 ± 0.004	8.83 × 10^−6^	1.587	1.228
Pyridines	2-Pentylpyridine ***	1572.4	2294-76-0	1.220 ± 0.270	0.468 ± 0.227	7.75 × 10^−4^	−1.383	1.101
	3-Ethylpyridine **	1378.6	536-78-7	0.068 ± 0.014	0.041 ± 0.011	1.76 × 10^−3^	−0.712	0.96
S-/N-Heterocycles	2-Furfuryl methyl sulfide **	1490.6	1438-91-1	0.652 ± 0.058	0.958 ± 0.268	2.21 × 10^−3^	0.555	0.885
	2-Ethyl-4-methylthiazole **	1350.7	15679-12-6	0.025 ± 0.002	0.019 ± 0.004	1.84 × 10^−3^	−0.387	0.941
	2-Acetylpyrrole	1963.1	1072-83-9	0.135 ± 0.019	0.112 ± 0.062	1.00 × 10^0^	−0.267	0.511
Amides/other	N,N-Dimethylacetamide(1)	1414.6	127-19-5	0.730 ± 0.109	0.732 ± 0.120	6.52 × 10^−1^	0.004	0.317
	N,N-Dimethylacetamide(2)	1414.6	127-19-5	0.553 ± 0.073	0.621 ± 0.087	1.16 × 10^−1^	0.167	0.530
	N,N-Dimethylformamide ***	1325.7	68-12-2	0.023 ± 0.003	0.047 ± 0.006	7.75 × 10^−4^	1.011	1.226
Unidentified	Unknown1 **		-	2.973 ± 0.262	2.567 ± 0.146	1.76 × 10^−3^	−0.212	0.932
	Unknown2 ***		-	0.173 ± 0.020	0.235 ± 0.030	4.14 × 10^−4^	0.443	1.030
	Unknown3 ***		-	0.032 ± 0.005	0.053 ± 0.004	3.41 × 10^−7^	0.721	1.217
	Unknown4 ***		-	0.359 ± 0.044	0.107 ± 0.009	5.93 × 10^−7^	−1.745	1.270
	Unknown5 ***		-	0.078 ± 0.014	0.039 ± 0.007	8.83 × 10^−6^	−0.991	1.139
	Unknown6		-	0.111 ± 0.018	0.130 ± 0.027	1.25 × 10^−1^	0.229	0.618
	Unknown7 ***		-	0.014 ± 0.002	0.044 ± 0.012	3.52 × 10^−4^	1.604	1.167
	Unknown8 *		-	0.097 ± 0.011	0.083 ± 0.010	1.47 × 10^−2^	−0.227	0.778
	Unknown9 ***		-	0.283 ± 0.013	0.123 ± 0.019	2.17 × 10^−11^	−1.204	1.275
	Unknown10 ***		-	0.185 ± 0.012	0.052 ± 0.015	2.17 × 10^−11^	−1.835	1.276
	Unknown11 *		-	0.042 ± 0.012	0.029 ± 0.009	3.61 × 10^−2^	−0.537	0.717
	Unknown12 ***		-	0.174 ± 0.031	0.046 ± 0.007	7.75 × 10^−4^	−1.928	1.232
	Unknown13 *		-	2.879 ± 0.109	2.778 ± 0.049	2.30 × 10^−2^	−0.051	0.701
	Unknown14 **		-	3.168 ± 0.094	2.882 ± 0.162	1.76 × 10^−3^	−0.136	0.998
	Unknown15 ***		-	0.483 ± 0.067	0.733 ± 0.021	7.75 × 10^−4^	0.601	1.215
	Unknown16		-	0.149 ± 0.029	0.222 ± 0.141	1.00 × 10^0^	0.570	0.613
	Unknown17		-	0.055 ± 0.019	0.058 ± 0.017	2.15 × 10^−1^	0.061	0.231

ZS, green (*Z. schinifolium*) and ZB, red (*Z. bungeanum*) Sichuan pepper essential oil. Data are mean ± SD (*n* = 9). q, FDR-adjusted p-value (*** q < 0.001, ** q < 0.01, * q < 0.05). log_2_FC (ZB/ZS): positive, higher in ZB; negative, higher in ZS. VIP, variable importance in projection. Some identified compounds that were represented by more than one signal in the experimental spectra have been retained as separate entries. To avoid potential ambiguity, these repeated entries are now labeled simply as “(1)” and “(2)”.
